# Phenotypic responses to microbial volatiles render a mold fungus more susceptible to insect damage

**DOI:** 10.1002/ece3.3978

**Published:** 2018-04-02

**Authors:** Silvia Caballero Ortiz, Monika Trienens, Katharina Pfohl, Petr Karlovsky, Gerrit Holighaus, Marko Rohlfs

**Affiliations:** ^1^ J.F. Blumenbach Institute of Zoology and Anthropology Animal Ecology Group University of Goettingen Goettingen Germany; ^2^ Molecular Phytopathology and Mycotoxin Research University of Goettingen Goettingen Germany; ^3^ Forest Zoology and Forest Conservation University of Goettingen Goettingen Germany; ^4^ Institute of Ecology, Population and Evolutionary Ecology Group University of Bremen Bremen Germany; ^5^ Present address: Institute for Evolution and Biodiversity University of Muenster Muenster Germany

**Keywords:** chemical interference, insect–fungus interactions, microbial ecology, multispecies interactions, secondary metabolites, volatile organic compounds

## Abstract

In decomposer systems, fungi show diverse phenotypic responses to volatile organic compounds of microbial origin (volatiles). The mechanisms underlying such responses and their consequences for the performance and ecological success of fungi in a multitrophic community context have rarely been tested explicitly. We used a laboratory‐based approach in which we investigated a tripartite yeast–mold–insect model decomposer system to understand the possible influence of yeast‐borne volatiles on the ability of a chemically defended mold fungus to resist insect damage. The volatile‐exposed mold phenotype (1) did not exhibit protein kinase A‐dependent morphological differentiation, (2) was more susceptible to insect foraging activity, and (3) had reduced insecticidal properties. Additionally, the volatile‐exposed phenotype was strongly impaired in secondary metabolite formation and unable to activate “chemical defense” genes upon insect damage. These results suggest that volatiles can be ecologically important factors that affect the chemical‐based combative abilities of fungi against insect antagonists and, consequently, the structure and dynamics of decomposer communities.

## 1 INTRODUCTION

Microorganisms produce a huge diversity of volatiles (Dickschat, 2017; Lemfack, Nickel, Dunkel, Preissner, & Piechulla, 2014) that cause phenotypic responses in other organisms. Because phenotypic responses alter species interaction outcomes (Agrawal, 2001), volatiles have the potential to indirectly generate variation in the abundance and distribution of organisms in multitrophic communities. For the study of community and food web ecology, it is therefore critically important to better understand the mechanisms and ecological consequences of phenotypic responses to volatiles.

Fungi are dominant inhabitants of decomposer systems and show striking phenotypic changes in response to volatiles, which include enhanced or reduced mycelial growth and aberrant morphologies, as well as altered enzyme activity, gene expression, and secondary metabolite formation (e.g., Effmert, Kalderás, Warnke, & Piechulla, 2012; Fialho et al., 2016; Mackie & Wheatley, 1999;Schmidt, Cordovez, de Boer, Raaijmakers, & Garbeva, 2015; Spraker et al., 2014). Fungi engage in a complex network of antagonistic and mutualistic interactions involving plants and myriads of invertebrates with fundamental impact on ecosystem functioning (Dighton, 2016). If the previously described phenotypic responses of fungi to volatiles affect traits relevant for the outcome of these interactions, the ecological function of fungi as food source, competitors, or pathogens, within the community context, may change.

Here, we ask whether phenotypic responses of a fungus to volatiles might influence the interaction outcome with an insect antagonist. The chemical phenotype of a fungus—as determined by its secondary metabolism—is central to the outcome of interactions with insects and other invertebrates. Repellent, deterrent, or toxic secondary metabolites are thought to have evolved as means to protect fungi against damage from invertebrate attack (Janzen, 1977). The biosynthesis of fungal “defense” chemicals is controlled by a complex molecular network involving protein kinase‐mediated signaling, transcriptional activation, and epigenetic modification (Keller, 2015). We therefore ask specifically if microbial volatiles influence the outcome of fungus–insect interactions by interfering with the mechanisms that regulate a fungus' chemical phenotype and thereby its capacity to resist insect damage.

**Figure 1:**
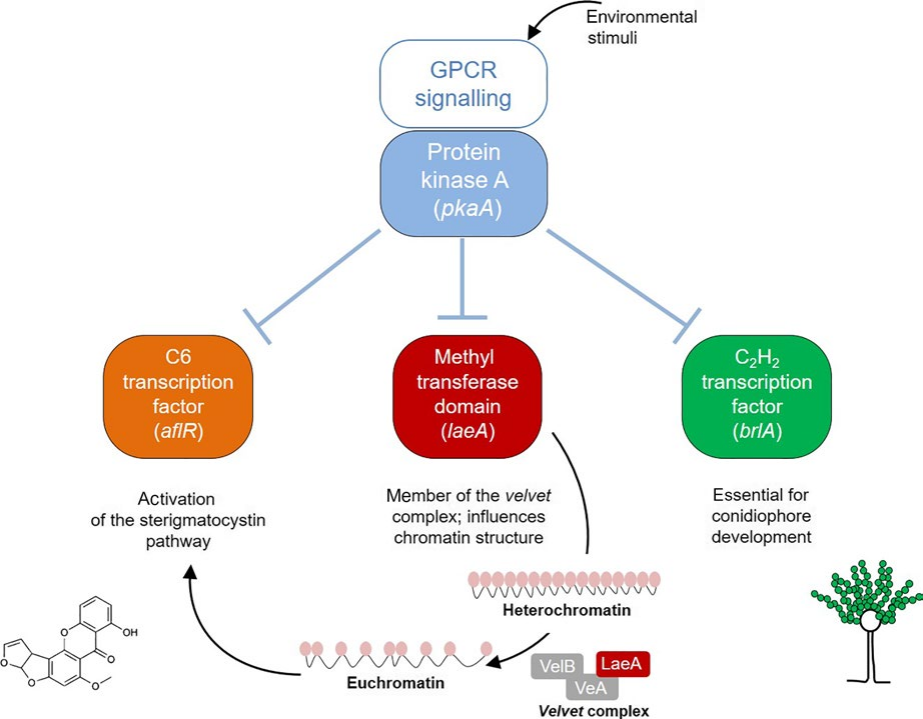
Schematic representation of molecular genetic elements involved in regulating morphological and chemical differentiation in *Aspergillus nidulans*. Ligands (mostly unknown) and G protein‐coupled receptors (GPCR) function in activating and deactivating cAMP‐dependent protein kinase A. High PkaA activity promotes vegetative growth, but inhibits morphological differentiation (e.g., asexual development) as well as secondary metabolite production. Asexual development is characterized by the formation of conidiophores and subsequent sporulation (see Figure 2c). The C_2_H_2_‐transcription factor BrlA is essential for the initiation and regulation of functional conidiophore development. The methyl transferase domain LaeA is part of the functional heterotrimeric velvet complex, involved in chromatin remodeling. A major consequence of LaeA‐mediated epigenetic changes is the activation of various secondary metabolite gene clusters, as indicated by the activation of pathway‐specific transcription factors, such as AflR from the sterigmatocystin cluster (Bayram & Braus, 2012). LaeA is also a major regulator of fungal resistance against insect damage. To test whether microbial volatiles interfere with this regulatory network of morphological and chemical differentiation in *A. nidulans*, we quantified volatile‐induced changes in candidate gene expression, *pkaA*,* brlA*,* laeA*, and *aflR*. See methods section for references and more details

We investigated a tripartite decomposer model system: the filamentous mold *Aspergillus nidulans* producing a wide range of insecticidal secondary metabolites, the yeast *Saccharomyces cerevisiae* as a volatile producer, and saprophagous dipteran larvae, *Drosophila melanogaster* (Figure S1). Growth and development of *D. melanogaster* larvae are bound to a wide variety of decaying fruits (Markow & O'Grady, 2008), on which diverse generalist molds and yeasts are dominant microbial inhabitants (e.g., Atkinson, 1981; Oakeshott, Vacek, & Anderson, 1989; Stamps, Yang, Morales, & Boundy‐Mills, 2012). Yeasts are an important food source for *Drosophila* larvae (Anagnostou, Dorsch, & Rohlfs, 2010). Larvae also readily graze on mold tissue, by which they temporarily suppress fungal colony growth, yet an induced chemical defense response (Caballero Ortiz, Trienens, & Rohlfs, 2013) turns mold fungi into allelopathic and rapidly expanding “weeds” that cause high mortality in fly larvae and reproductive dysfunctions in those individuals that reach the adult stage (Hodge, Mitchell, & Arthur, 1999; Wölfle, Trienens, & Rohlfs, 2009; Figure S1). Experiments using mutant fungi showed that the mold's insecticidal properties are linked to their secondary metabolism (Trienens, Keller, & Rohlfs, 2010) (Figure 1). The insecticidal properties are strong in strictly bipartite mold–larval interactions where other microbes were explicitly excluded (Trienens et al., 2010; Caballero Ortiz et al., 2013), whereas under more natural conditions that include the metabolic activity of yeasts, *Drosophila* larvae can hinder or even permanently suppress mold growth (Stamps et al., 2012; Rohlfs & Hoffmeister, 2005; Wertheim, Marchais, Vet, & Dicke, 2002). This latter observation prompted us to assume that in our tripartite model system insects are better able to keep mold in check because *S. cerevisiae* volatile metabolites interfere with the molecular and chemical mechanisms by which *A. nidulans* harms the insects in the absence of these volatiles.

**Figure 2:**
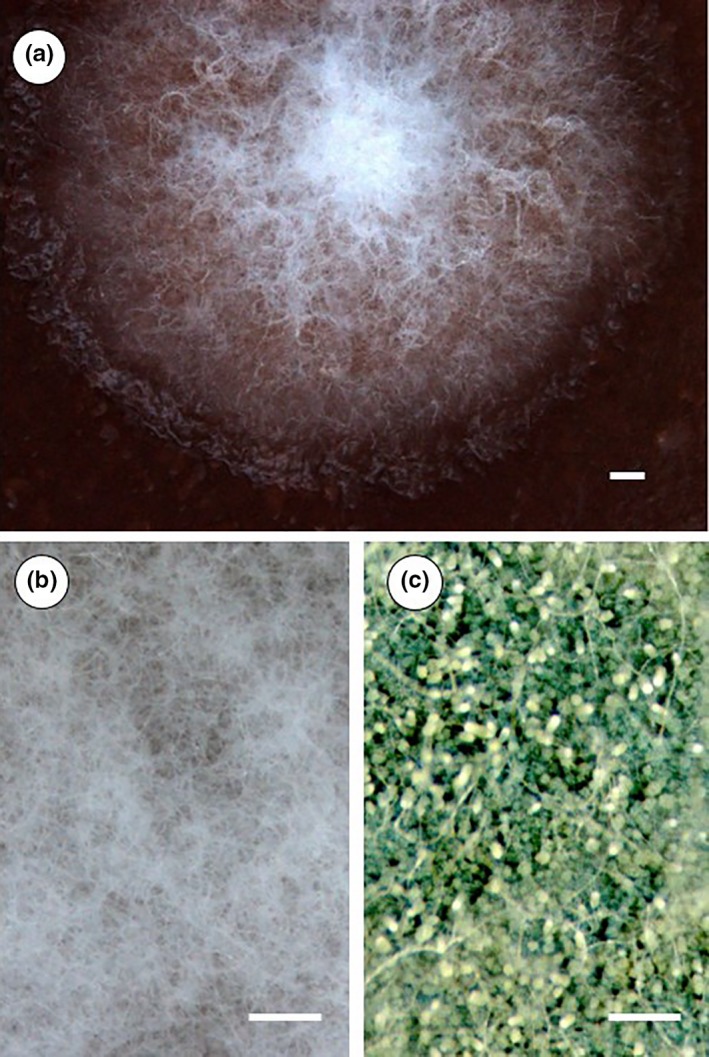
Yeast volatiles influence morphological differentiation of *Aspergillus nidulans*. Mold development under the influence of volatiles produced by *Saccharomyces cerevisiae* on naturally sugar‐rich or sucrose‐supplemented medium results in colonies with a densely packed hyphal tissue and numerous aerial hyphae (a, b). *Aspergillus nidulans* readily formed conidia in a nonvolatile treatment or when exposed to volatiles the yeast produced on artificial medium without additional sucrose (c)

In this experimental study, *A. nidulans* exposed to yeast volatiles showed systemically altered colony growth characterized by a lack of conidiospore development (Figure 2). Strikingly, we found this change in the mold's morphological phenotype only in a carbohydrate‐rich environment, that is, fruit or sugar‐supplemented culture medium. To gain insights into how these volatile‐mediated changes in mold morphology are related to the molecular and chemical phenotype and feed back on the interaction outcome with the insects, we characterized the yeast volatile chemistry and ran a series of experiments to test: (1) whether the altered morphology of *A. nidulans* shows changes in the expression of genes involved in signal transduction, regulation of conidiation and resistance to insect damage (Figure 1), (2) whether the outcome of the mold–insect interaction is shifted in favor of the fly larvae, (3) whether the inducibility of insecticidal defense marker genes by larval grazing is suppressed, and (4) whether the chemical phenotype of *A. nidulans* is affected as well. By establishing a causal link between microbial volatile emissions and changes in fungal resistance to insect damage, we argue that incorporating phenotypic responses to volatiles may advance our functional understanding of variation in the outcome of decomposer species interactions.

## 2 MATERIALS AND METHODS

### 2.1 Culture of organisms

*Aspergillus nidulans* (RDIT1.3) and *S*. *cerevisiae* (DSM70449) were cultured following standard protocols (Anagnostou et al., 2010; Caballero Ortiz et al., 2013). Active cultures were started from glycerol stocks stored at −80°C. For each experiment described below, we started mold cultures with inoculating suspensions adjusted to 1,000 conidiospores per microliter and yeast cultures with suspensions adjusted to 1,500 cells per microliter. These suspensions were prepared in sterile Ringer solution (8.6 g NaCl, 300 mg KCl, 350 mg CaCl_2_ per liter deionized water) containing Tween 80 (0.1%).

The *D. melanogaster* rearing originated from a field population collected in 2006, in Kiel, northern Germany. The population was maintained as a mass culture according to our established protocol (Trienens et al., 2010). To obtain larvae for the experiments described below, we allowed flies to lay eggs in culture flasks for ~12 hr. We sterilized the eggs to avoid contamination of the experiments with unwanted microbes attached to the eggs or the egg‐laying substrate. This involved dechorionating the eggs with 6% sodium hypochlorite, thoroughly washing them with sterile water, and incubating them overnight in Petri dishes containing sterile water agar at 25°C (Trienens et al., 2010).

### 2.2 Yeast–mold coculture experiments

In preliminary experiments, we grew *S. cerevisiae* on various culture media, to test whether the yeast volatiles produced on these media differentially influence mold growth and morphology. *Drosophila* culture medium (Trienens et al., 2010), banana medium (Anagnostou et al., 2010), and standard malt extract agar with and without sucrose were tested. With experiments that controlled for any effects of physical contact or diffusion of nonvolatile compounds, we found the yeast volatile emissions to suppress mold growth and conidiation in all substrates, except for malt extract agar with no sucrose added (see Supporting information “Yeast volatile‐mediated effects on mold growth in different culture media”). These results suggest that the amount of sugar available in the growth medium regulates the yeast's ability to release mold‐affecting volatiles.

To mimic a fruit‐like sugar‐rich habitat without the variability of using real fruit, we used malt extract agar (30 g standard malt extract [Carl Roth, X976.x, Germany], 5 g soy peptone [Carl Roth, 2365.x, Germany], and 20 g agar–agar [Kobe I, 5210.x, Carl Roth, Germany] in a liter of water). For the sugar‐rich treatment, we supplemented this medium with D‐sucrose (Carl Roth, 4621.x, Germany) at 60 g/L. The resulting sucrose concentration is within but at the low end of the range for sugars in fruits (Lievens et al., 2014). Therefore, it does not overestimate the role of sugar in our model system compared to that in nature. The details of the coculture experiments are described in the Supporting information “Yeast volatile‐mediated effects on mold growth in different culture media”.

### 2.3 Yeast volatile analysis

To test whether the substrate‐dependent effects of yeast on mold morphology and growth are related to qualitative and/or quantitative differences in the composition of volatile metabolites emitted by *S. cerevisiae*, we cultivated the yeast on both type of media (“no sucrose” vs. “plus sucrose”) and analyzed the volatiles emitted from the yeast colonies. For this, thoroughly cleaned glass vials of 65 ml volume (13.3 cm height, 2.5 cm diameter) were filled with 20 ml of the respective malt extract agar. The vials were covered with aluminum foil and autoclaved. After hardening of the agar, the substrate was inoculated with 50 μl yeast cell suspension. Eight vials per treatment were prepared and incubated for 4 days at standard climatic conditions (25°C, 12‐hr light cycle). Subsequently, we extracted the headspace volatiles by means of solid phase micro‐extraction (HS‐SPME). The fiber type (85 μm CarboxenTM/PDMS StableFlexTM) and a sampling time of 5 min at 20°C were chosen based on previous studies on fungal volatiles (Nilsson, Larsen, Montanarella, & Madsen, 1996). Identification of volatile compounds by GC‐MS followed established protocols (see Stötefeld, Holighaus, Schütz, & Rohlfs, 2015), detailed in the Supporting information “Specifications of the GC‐MS analysis of yeast volatiles.”

For the quantification of volatiles, extracted ion chromatograms (EIC) were integrated manually and recalculated to peak areas of total ion chromatograms (TIC) based on the EIC:TIC ratio of the standard compounds. Peak areas were scaled to their mean (*z*‐score). To describe sucrose‐mediated changes in yeast volatile formation, we created Venn diagrams for depicting presence/absence of volatiles as a function of treatment, subjected the scaled areas to a hierarchical heat‐map clustering analysis, and calculated fold changes in volatile formation using MetaboAnalyst, http://www.metaboanalyst.ca (Xia, Sinelnikov, Han, & Wishart, 2015). To further confirm the differences of the volatile profiles, we applied a machine learning algorithm (random forest analysis), to test whether the samples can be correctly assigned to predefined treatment groups (“no sucrose” or “plus sucrose”) (Breiman, 2001) based on volatile metabolites (peak areas) in a number of iterations. We used the “randomForest” package implemented in R (R Core Team, 2012), which returns a confusion matrix that contains a summary of the classification procedure and the mean prediction error, the “out‐of‐bag” error. We drew 10,000 bootstrap samples (trees) with three randomly selected variables at each node. The random forest analysis also provides a measure of importance of individual variables for a correct classification, the “mean decrease accuracy” (MDA) value. Variables that contribute strongly to the correct classification receive high positive MDA values. Small MDA values contribute little to it and negative values counteract a correct classification.

### 2.4 Fungus–insect fitness consequences under the influence of yeast volatiles

Experimental units designed to allow diffusion of yeast volatiles, while keeping insect larvae in a mold compartment (Figure S2), were used to test whether yeast volatiles can affect the outcome of the mold–insect interaction. The yeast was inoculated on “plus sucrose” malt extract agar to stimulate the release of mold‐affecting volatiles. On the opposite side, mold conidia were point‐inoculated on banana medium; note that on this medium the *A. nidulans* wild‐type strain causes 100% larval mortality, and in the absence of beneficial microbes larvae do not develop at all, see Caballero Ortiz et al. (2013) and Anagnostou et al. (2010). The mold grew for 6 days in the presence of the *S. cerevisiae* volatiles, which resulted in the same kind of aconidial colonies as observed in the previously described experiments (see Supporting information “Yeast volatile‐mediated effects on mold growth in different culture media”). On day six, we added ten first‐instar *D. melanogaster* larvae per experimental unit to the mold compartment. To quantify the effect of the mold on *Drosophila* developmental success, emerging adult flies were recorded and removed according to a daily scheme until day 24 after the transfer of the larvae. This setup was replicated 17 times. In parallel, we ran the same number of replicates without mold to check for any systematic error like infestation of the mold compartment with *S. cerevisiae* or other microbes that could have caused development of the fly larvae on the banana agar. Again, banana agar does not support larval development unless beneficial microbes are added (Anagnostou et al., 2010), which we verified for the abovementioned setup by exposing larvae to mold without the influence of yeast volatiles (*n* = 5). Here, the opposite compartment contained microbe‐free malt extract agar.

To quantify the influence of larval activity on volatile‐exposed mold, that is, whether *A. nidulans* colonies recover from larval activity—as they would normally do when not exposed to yeast volatiles (Trienens et al., 2010)—we set up the same experiment as described above, with five replicates with and without larvae. We started imaging the mold compartment after 72 hr and continued taking photos according to a daily scheme until day 11 (264 hr) after inoculation. On day six (144 hr postinoculation), we added ten larvae to five units randomly assigned to the insect treatment. The images were used to determine the area covered (in mm²) by mold and hence changes in mold growth as a function of time (see Supporting information “Yeast volatile‐mediated effects on mold growth in different culture media” for more details). To test for significant changes in fungal colony size over time, we applied a general estimation equation (GEE) model (geeglm in R; R Core Team, 2012) with “larval treatment” and “time” as a fixed and “colony identity” as a random factor.

### 2.5 Mold gene expression analysis

The investigation of gene expression in *A. nidulans* had two purposes: (1) to explore the influence of yeast volatiles on key molecular regulators of morphological and chemical differentiation of *A. nidulans*; and (2) to test whether volatile‐exposed mold loses its capacity to activate candidate “insecticidal defense” genes in response to insect damage (Caballero Ortiz et al., 2013). The expression of fungal secondary metabolite regulator genes was measured. These were genes encoding the nuclear protein LaeA, a member of the Velvet complex controlling global secondary metabolite biosynthesis (Bok & Keller, 2004), and AflR, a transcription factor involved in the activation of the sterigmatocystin mycotoxin pathway (Fernandes, Keller, & Adams, 1998). As an indicator of aberrant morphogenesis, the expression of *pkaA* was quantified. PkaA is an important downstream element of G protein‐coupled receptor‐mediated signaling and plays a key role in activation/repression of vegetative growth, conidiation, and secondary metabolite production (Yu & d'Enfert, 2008). We also examined the expression of *brlA* which encodes for a transcription factor required for conidiation (Adams, Boylan, & Timberlake, 1988). See Figure 1 for details and interconnections of these pathways.

Purpose (i): *A. nidulans* colonies were prepared by spreading 100 μl conidia suspension on autoclaved cellophane foil placed on sucrose‐supplemented malt extract agar in 35‐mm plates. The foil allows diffusion of nutrients without affecting mold growth (Caballero Ortiz et al., 2013), but ensures sampling of pure fungal tissue without medium contaminations. The full fungal tissue was harvested with a scalpel. Coculture of mold and yeast followed the same procedure as described in the Supporting information (see above). Because cocultivation arrested *A. nidulans* in a developmental stage shortly before the fungus starts producing conidial heads (see Figure 1b,d in the Result section), we decided to sample 2‐day‐old colonies as appropriate nonexposed controls. Under the given culture conditions, colonies at that age were about to produce aerial hyphae that developed conidial heads at their tips during the upcoming 24 hr. At these stages, the fungal tissue was harvested with a scalpel, shock‐frozen in liquid nitrogen, and lyophilized. Isolation of RNA from defined amounts of lyophilized *A. nidulans* tissue (100 mg per replicate), RNA quality checks, PCR conditions, and data analysis followed the protocol described in Caballero Ortiz et al. (2013) (for more details and primers used see Supporting information “Gene expression analysis”).

Purpose (ii): *A. nidulans* colonies were cocultivated with *S. cerevisiae* in a shared airspace chamber as described previously. After 6 days, 40 first‐instar *D. melanogaster* larvae that hatched from sterilized eggs were transferred to individual *A. nidulans* colonies. Larvae could graze for 24 hr and were then removed and the insect‐damaged fungal tissue was harvested and treated as described above for the transcriptomic analysis.

### 2.6 Mold secondary metabolite analysis and identification

Five seven‐day‐old colonies of *A. nidulans* exposed to yeast volatiles and five nonexposed colonies were used to search for chemical differences developed under these conditions. As for the gene expression analysis, the colonies were grown on cellophane placed on culture medium (see above). Lyophilized, ground mycelium was extracted with acetonitrile–water (84/16) of ten times the sample weight. The extracts were cleared by centrifugation, the solvent was removed in vacuum, and the residues were dissolved in methanol–water (1:1) and defatted with cyclohexane.

Nontargeted chemical analysis was conducted by HPLC coupled with an ion trap mass spectrometer equipped with an electrospray ionization source (HPLC‐ESI‐MS). An RP column eluted with a water/methanol gradient was used as described by Ratzinger, Riediger, von Tiedemann, and Karlovsky (2009). The ion trap was operated in positive mode under conditions described by Khorassani et al. (2011). Raw data were processed with a component detection algorithm (Windig, Phalp, & Payne, 1996) implemented in the ACD/MS Manager version 12.0 (Advanced Chemistry Development, Toronto, Canada) with an MCQ (mass chromatography quality) threshold of 0.8 and a window width of three scans (see Chatterjee, Kuang, Splivallo, Chatterjee, & Karlovsky, 2016 for details). For peak picking, signals with intensities of less than 500,000 counts and/or signal‐to‐noise ratios of less than 100 were discarded. The resulting peak tables were processed using custom Perl scripts for peak alignment and normalization (Laurentin, Ratzinger, & Karlovsky, 2008).

Targeted HPLC‐MS/MS analysis was used for the identification and quantification of sterigmatocystin (*m*/*z* 325 to 281 and *m*/*z* 325 > 310), austinol (m*/*z 459 > 441 and *m/z* 459 > 423), dehydroaustinol (*m/z* 457 to 439 and *m/z* 457 to 421), emericellamide C/D (*m*/*z* 596 to 525 and m/z 596 to 507)*,* and emericellamide E/F (*m*/*z* 624 to 553 and *m/z* 624 to 464). The ion trap was operated in a positive ionization mode with the following source conditions: needle voltage +5,000 V, shield voltage +600 V, capillary voltage +100 V, drying gas (nitrogen) 15 psi at 350°C, and nebulizing gas (air/nitrogen) 25 psi. Data were acquired with a scan speed of 15,000 Da/s. The identity of target compounds was confirmed by at least two mass transitions. We used the same statistical routines (Venn diagram, clustering, random forest) as for the analysis of yeast volatiles to describe the chemical differences between the unexposed and volatile‐exposed colonies.

## 3 RESULTS

### 3.1 Yeast volatiles affected mold morphological differentiation and gene expression

The mold–yeast coculture assays revealed two kinds of phenotypic changes in the mold that were also visible by eye. First, under the influence of yeast volatiles *A. nidulans* formed hyphal mats that did not produce conidia (Figure 2). Further, radial growth of point‐inoculated *A. nidulans* colonies was significantly reduced in the presence of the yeast volatiles. After 7 days, colonies were only about 1/6 the size of colonies grown under yeast‐free conditions (Figure S3). Notably, this mold phenotype was found only when the cocultured yeast grew in naturally sugar‐rich medium or standard culture medium supplemented with sucrose (Figure S3).

**Figure 3:**
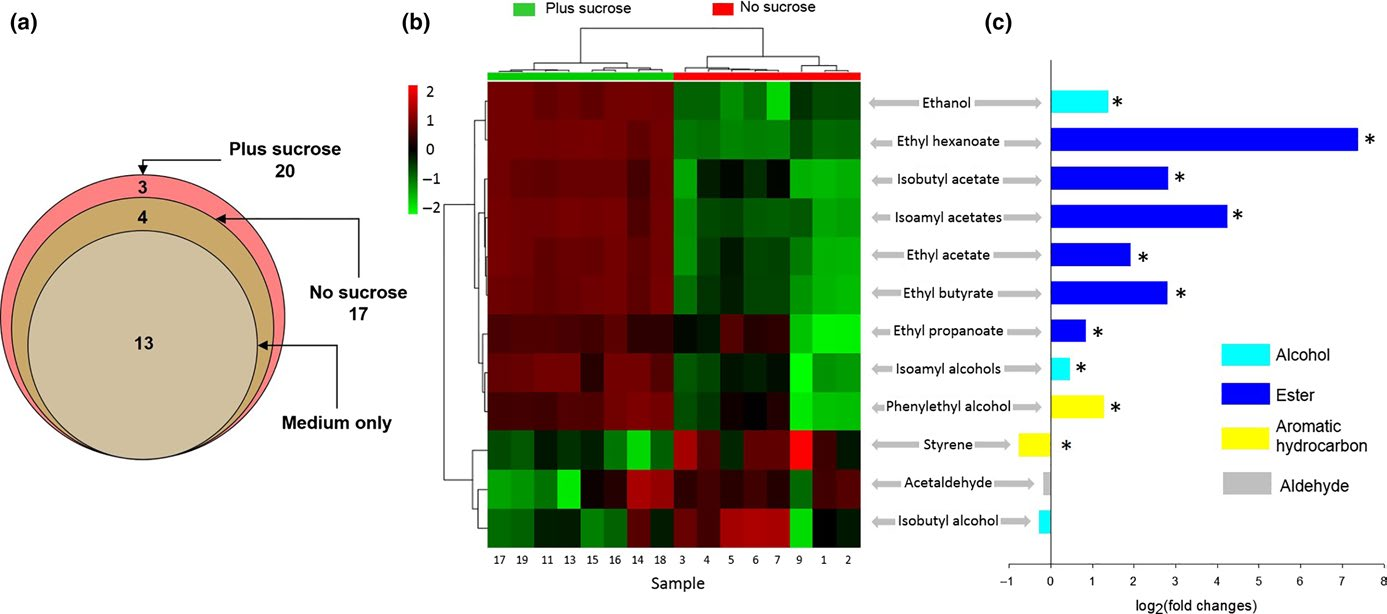
Sucrose‐mediated shifts in the volatile profile of *Saccharomyces cerevisiae*. a) Venn diagram depicting presence and absence of volatiles emitted by yeast growing on no and plus sucrose malt extract agar, and the medium without yeast. b) Heat‐map analysis and hierarchical clustering (Ward's method, Euclidean distance) of the twelve compounds found in all yeast samples, excl. those that were mainly emitted by the medium alone. c) Sucrose‐mediated fold changes in yeast‐specific volatiles. Asterisks depict significant changes based on false discovery rate (FDR)‐adjusted *p* ≤ .05. The “isoamylalcohols and isoamylacetates” each comprise two compounds, 3‐methyl‐1‐butanol and 2‐methyl‐1‐butanol, and 3‐methyl‐1‐butanol acetate and 2‐methyl‐1‐butanol acetate, respectively. They entered the statistical analyses as one compound each as their quantities could not properly be determined (Figure S4). See text and Table S1 for more information and statistical details

We found striking differences in the volatile profiles produced by *S. cerevisiae* in sucrose‐deficient and sucrose‐supplemented medium (Figures 3 and S4). Seven volatile compounds appeared exclusively in the yeast treatments, of which three compounds were found only when yeast grew in the sucrose‐supplemented variant (Figure 3a). Some of the minor compounds emitted by the medium were even less abundant in the yeast treatment (Table S1). We excluded these compounds from the characterization of the mold‐affecting volatile profile, as we did not expect a reduction in nonyeast volatiles to influence *A. nidulans*. A subsequent cluster analysis for the yeast‐specific volatiles showed perfect clustering of the volatile samples according to the plus/no sucrose treatment (Figure 3b). Correspondingly, the random forest analysis correctly assigned all the volatile profiles to the plus/no sucrose treatment (out‐of‐bag error = 0%).

The mold‐affecting profile produced on sucrose‐supplemented medium is characterized by significantly larger amounts of ten compounds—six esters, ethanol, two amylalcohols (3‐methyl‐1‐butanol, 2‐methyl‐1‐butanol), phenylethylalcohol—plus the de novo occurrence of ethyl octanoate, ethyl decanoate, and phenylethyl acetate (Figure 3c). The extent of fold changes was clearly compound‐specific with ethyl hexanoate showing the strongest increase (Figure 3c). However, according to the random forest analysis, almost all sucrose‐induced compounds contributed equally to the classification of the yeast volatile profiles (Table S1).

**Figure 4:**
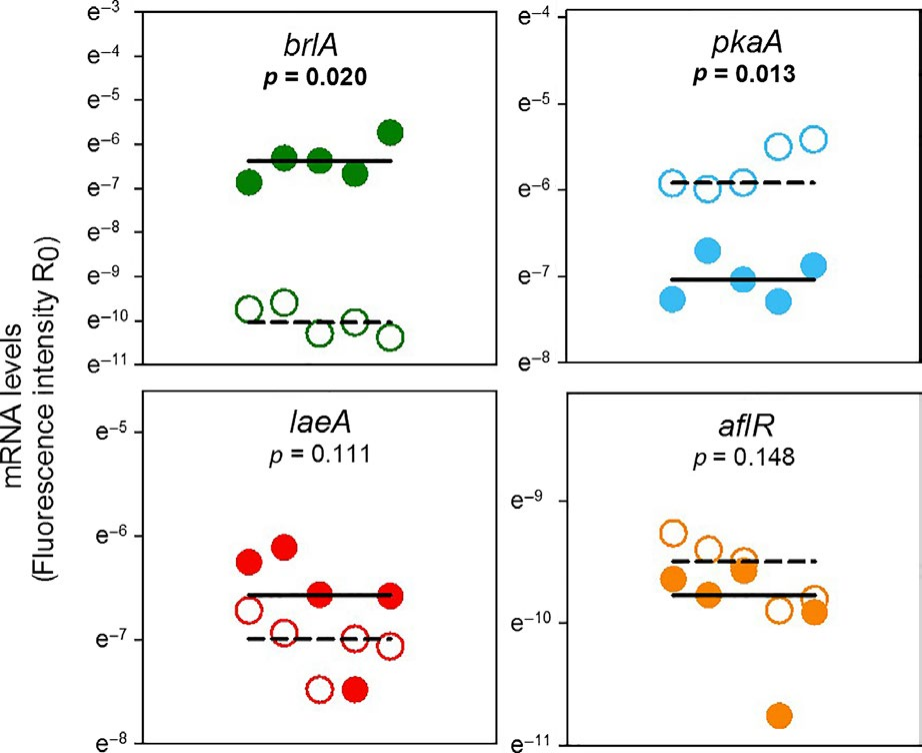
Gene expression in *Aspergillus nidulans* as affected by yeast volatiles. FDR‐adjusted *p*‐values represent the results of post hoc univariate ANOVAs on ranks, following a MANOVA (see text). Gene names and colors represent those shown in Figure 1. Solid and dashed lines depict the medians for the gene expression levels in nonexposed (open symbols) and volatile‐exposed (filled symbols) mold, respectively

To explore the molecular mechanisms of the morphological differences between exposed and unexposed *A. nidulans* and its possible connection with changes in the regulation of insecticidal properties, we quantified the transcription levels of *pkaA*,* brlA*,* laeA*, and *aflR*. The corresponding gene products have central roles in major pathways controlling *A. nidulans* morphogenesis and secondary metabolism (see Figure 1 and Method Section). Volatiles from *S. cerevisiae* induced significant changes in the gene expression profile of the four genes (Figure 4; MANOVA, Pillai's trace = 0.97, *F*_4,5_ = 42.90, *p* < .001). Specifically, volatile‐exposed *A. nidulans* expressed more of the “signaling” gene *pkaA* (on average 3.6‐fold) and less of the “conidiation” gene *brlA* (on average 38.5 fold), than did nonexposed *A. nidulans*. Volatiles had only little effect on the expression of the global “secondary metabolite regulator” gene *laeA* or the “pathway‐specific regulator” gene *aflR* (Figure 4).

### 3.2 Mold phenotypic responses to yeast volatiles changed the outcome of interaction with insect larvae

**Figure 5:**
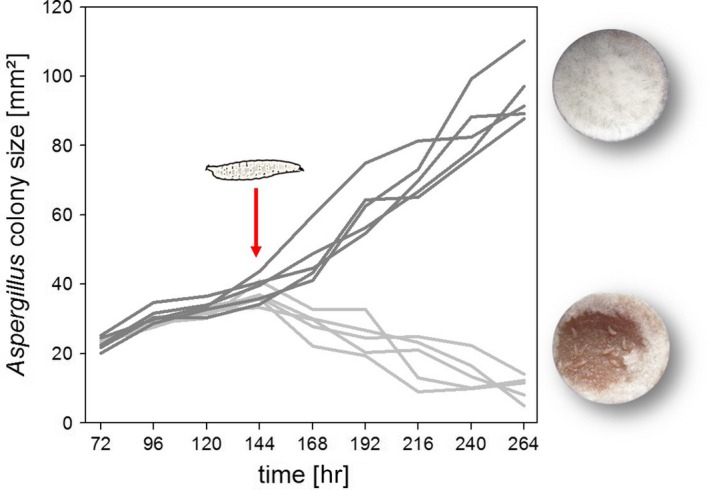
Impact of *Drosophila melanogaster* larvae on volatile‐exposed *Aspergillus nidulans*. Dark gray lines depict the growth (in mm² medium covered by mold mycelium) of five independent mold colonies when exposed to yeast volatiles only. The light gray lines represent the growth of five volatile‐exposed mold colonies that were, after an incubation period of 144 hr, exposed to fly larvae. The GEE model revealed that from hour 144 onwards time‐dependent mold growth was significantly affected by larval activity; there was a significant effect of larval treatment (χ² = 550, *df* = 1, *p *<* *.001), indicating lower overall mold growth for the larval treatment relative to the nonlarval treatment. There was also a significant effect of the interaction between time and larval treatment, indicating different slopes in mold growth (χ² = 270, *df* = 1, *p *<* *.001). While without larvae *A. nidulans* colony size increased linearly (regression equation: *colony size* = −29.96 + 0.47 *× *time), larval activity caused a linear decline (regression equation: *colony size* = 66.95 − 0.22 *× *time). Including higher order terms for “time” did not improve the model, so there was no nonlinear change in mold growth in the period from the introduction of the larvae and the end of the observation

To test if yeast volatiles affect the *A. nidulans*–*D*. *melanogaster* interaction, we quantified both larval development on mold colonies to the adult stage and suppression of mold growth by foraging larvae. Two major effects could be observed: First, no larvae survived on unexposed mold, which verifies previously made observations (Caballero Ortiz et al., 2013); however, on volatile‐exposed mold, almost 50% (mean 4.88 ± 1.32 *SD* of ten larvae per replicate; *n* = 17) of the *D. melanogaster* larvae developed into adult flies. This indicates that volatile‐exposed mold lost its insecticidal properties. Larval development was not supported by volatiles alone. Second, larvae greatly suppressed the growth of volatile‐exposed *A. nidulans*. Moreover, as indicated by a linear decrease in mold cover in the insect treatment (Figure 5), the volatile‐exposed mold did not recover from larval damage, although it very quickly does so if not exposed to yeast volatiles (Caballero Ortiz et al., 2013; Trienens et al., 2010).

**Figure 6:**
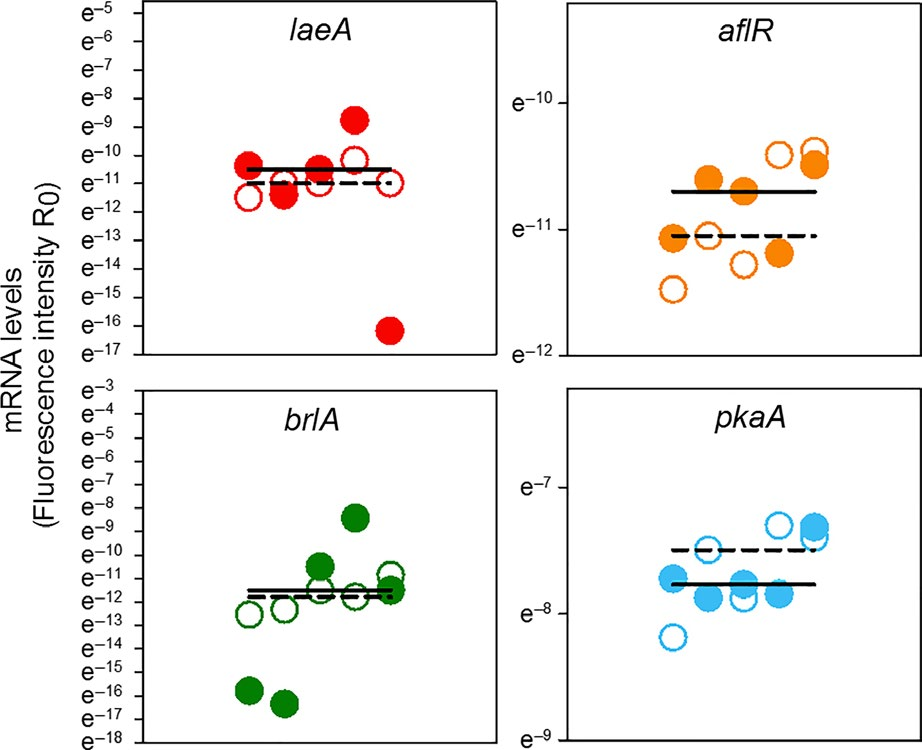
Gene expression in volatile‐exposed *Aspergillus nidulans* as affected by larval *Drosophila melanogaster* activity. Gene names and colors represent those shown in Figure 1. Solid and dashed lines depict the medians for the gene expression levels in nonconfronted (filled symbols) and insect‐confronted (open symbols) mold, respectively

The volatile‐mediated shift from the insecticidal to the noninsecticidal phenotype of *A. nidulans* is linked to a blockage of the molecular mechanisms mediating inducible resistance against insect. The unexposed/insecticidal *A. nidulans* phenotype has been shown to upregulate *laeA, aflR*, and *brlA* in response to larval grazing (Caballero Ortiz et al., 2013). In contrast to this previous observation, the volatile‐exposed/noninsecticidal *A. nidulans* phenotype did not respond with a change in expression of these genes to insect damage (Figure 6; MANOVA, Pillai′s trace = 0.23, *F*_4,5 _= 0.38; *p* = .817).

### 3.3 Yeast volatiles affected the diversity of *A. nidulans* metabolites

**Figure 7:**
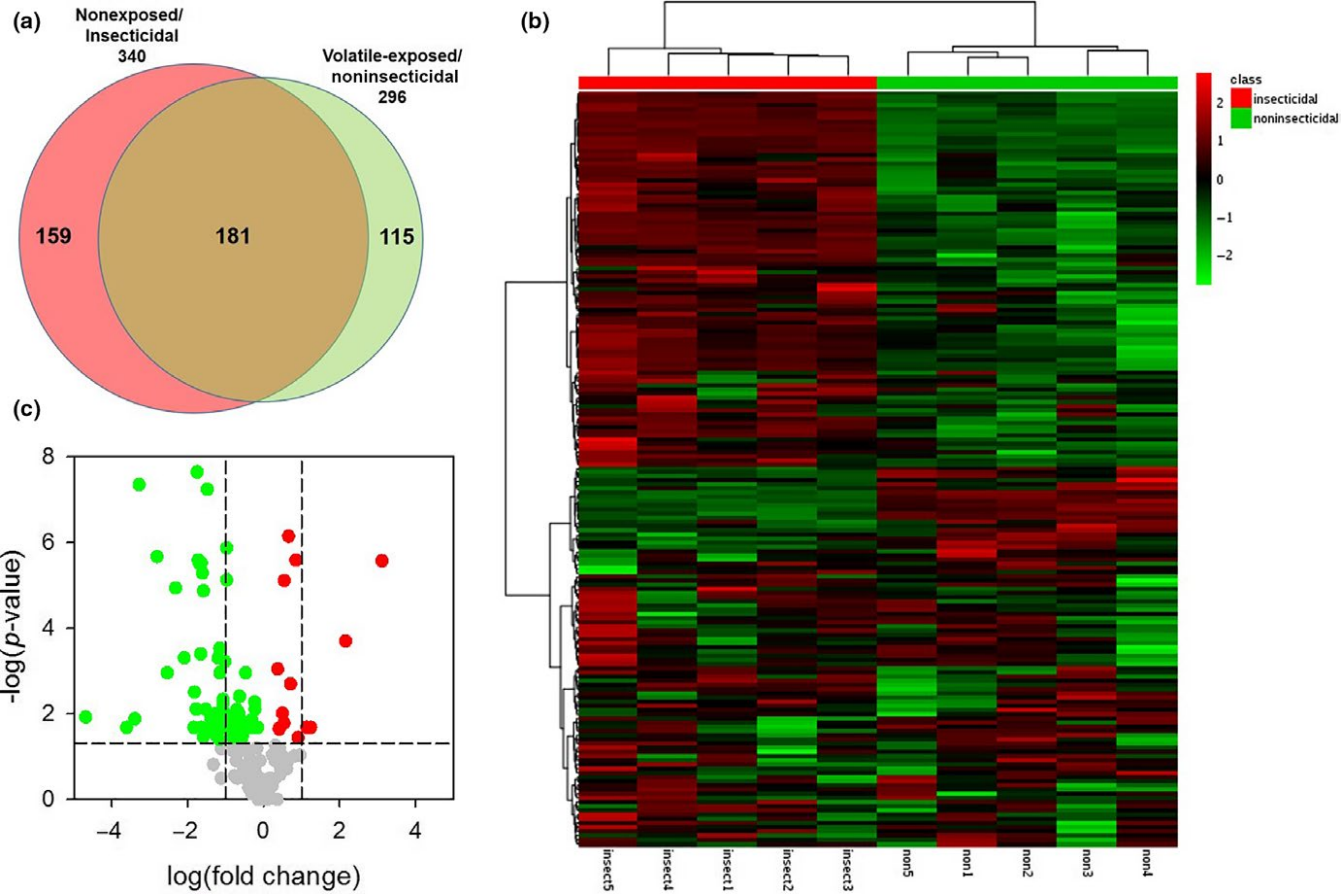
Chemical differentiation between nonexposed/insecticidal and volatile‐exposed/noninsecticidal *Aspergillus nidulans* as determined by HPLC‐ESI‐MS. a) Venn diagram depicting presence and absence of masses in insecticidal and noninsecticidal mold phenotypes. b) Heat‐map analysis and hierarchical clustering (Ward's method, Euclidean distance) of the 181 masses shared between the insecticidal and the noninsecticidal mold phenotype. c) Volcano plot, ‐ log10(FDR‐adjusted *p*‐values) against log2(fold changes), representing differences in the formation of 181 masses shared by insecticidal and noninsecticidal mold phenotypes. Green and red dots indicate masses significantly down‐ and upregulated, respectively, in the noninsecticidal mold phenotype. Vertical lines indicate the cutoffs for twofold changes. The horizontal line depicts the cutoff for statistical significance (FDR‐adjusted *p *≤* *.05)

To confirm that the suppression of fungal secondary metabolism contributed to the noninsecticidal phenotype of *A. nidulans*, we screened fungal extracts for changes in chemical composition by HPLC‐ESI‐MS. The HPLC profiles revealed a striking decline in chemical diversity in *A. nidulans* colonies exposed to volatiles. HPLC profiles of extracts from colonies exposed to volatiles lost about 47% of the 340 signals found in unexposed colonies. Interestingly, we found 115 new signals occurring only in colonies exposed to volatiles (Figure 7a). A cluster analysis based on the intensity of the 181 signals shared by the volatile‐exposed/noninsecticidal and the unexposed/insecticidal phenotypes showed perfect clustering of samples according to the treatments (Figure 7b). The result of a random forest analysis strongly supports this classification as all mass profiles were correctly assigned to the treatments (overall out‐of‐bag error = 0%). Many of the signals found in both phenotypes were significantly downregulated in the noninsecticidal phenotype (37.5% in total; among them 24% more than twofold), while only few signals (7.7%) were significantly upregulated (Figure 7c, Table S2).

**Figure 8:**
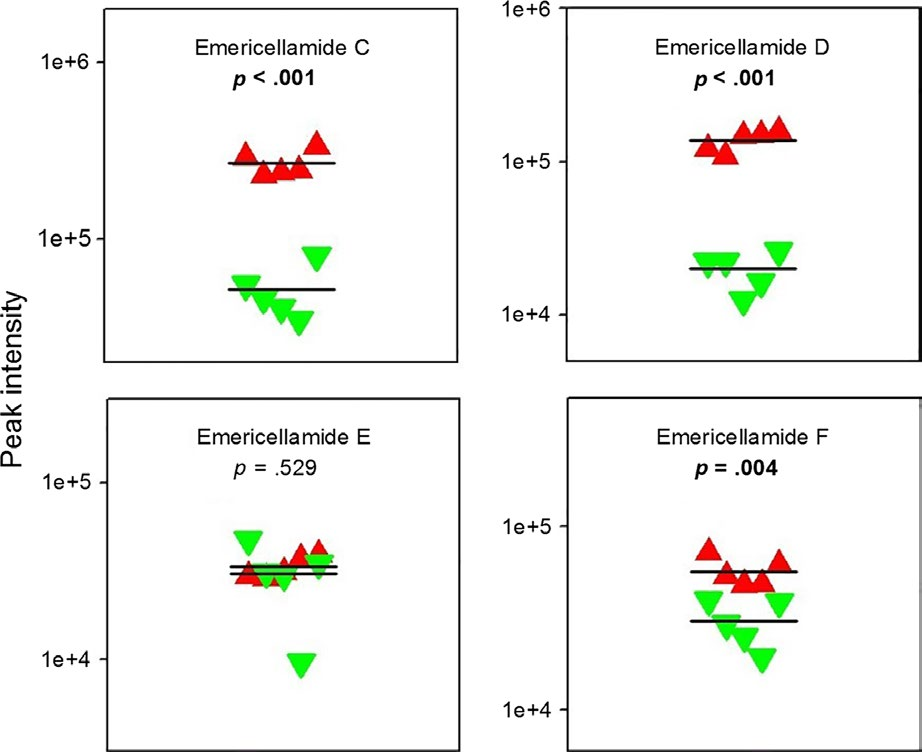
Peak intensities of metabolic signals indicative of the emericellamides. Red symbols (up‐pointing triangles) represent signal intensities from nonexposed/insecticidal, and green symbols (down‐pointing triangles) from volatile‐exposed/noninsecticidal *Aspergillus nidulans* phenotypes for each independent replicate. FDR‐adjusted *p*‐values show the results of two‐sided *t*‐tests. Horizontal lines depict means

Targeted analysis of selected secondary metabolites with potential roles in the defense against insects showed a significant downregulation of emericellamides in the volatile‐exposed/noninsecticidal phenotype (Figure 8). Meroterpenoids austinol and dehydroaustinol, which are abundant in the mycelium of the insecticidal *A. nidulans* phenotype, were no longer detectable in *A. nidulans* colonies exposed to volatiles (Figure S5). In previous studies, we found these compounds and their underlying biosynthetic genes upregulated in response to insect damage, which suggests their contribution to the insecticidal properties of the mold (Döll, Chatterjee, Scheu, Karlovsky, & Rohlfs, 2013; Caballero Ortiz et al., 2013).

## 4 DISCUSSION

Our results show that volatiles can influence the combative abilities of fungi and, consequently, the outcome of interactions with insects. The effect of this indirect interaction between volatiles and the insects is shown by an almost 50% reduction of fruit fly larval mortality in the presence of volatile‐exposed *A. nidulans*. Because unexposed mold caused 100% larval mortality, yeast volatiles seem to be essential to turn the mold‐substrate matrix into a suitable insect habitat. This finding demonstrates the enormous potential of volatiles to affect species interactions. Moreover, yeast volatiles formed on sugar‐rich substrate and larval foraging activity appear to be an effective combination of microbial chemical and insect behavioral factors that protects the insect breeding habitat from being overgrown and dominated by the allelopathic mold. This indirect and context‐dependent effect of microbial volatiles in our model system may at least in part explain the previously observed inverse relationship between mold growth and insect development on decaying fruits (Hodge, 1996; Stamps et al., 2012; Wertheim et al., 2002; Rohlfs & Hoffmeister, 2005). Therefore, volatiles by eliciting phenotypic responses in fungi can indirectly affect the relative dominance of interacting fungal and animal saprophages and hence community properties, for example, decomposer food web structure.

The loss of insecticidal properties in the volatile‐exposed *A. nidulans* phenotype coincided with down‐ and upregulation of *brlA* and *pkaA*, respectively, which indicates that the mold was halted in a vegetative stage unable to initiate the molecular processes required for conidiophore development (see Figure 1). Enhanced transcription of *pkaA* may be indicative of a more active cAMP/PkaA signaling cascade, which is correlated with reduced *brlA*‐dependent conidiation in a *pkaA* overexpression mutant (Shimizu & Keller, 2001). In addition to the blockage of morphological differentiation, artificial overexpression of *pkaA* has been shown to also suppress transcription of the secondary metabolite regulator *laeA* (Bok & Keller, 2004). We thus postulate that volatile‐mediated upregulation of *pkaA* sustainably prevents insect‐induced secondary metabolite formation in *A. nidulans* and thereby influences the chemical environment to which *D. melanogaster* larvae are exposed to.

The global loss of chemical diversity in volatile‐exposed *A. nidulans* is at least in part the result of suppressed *laeA*‐depended mechanisms necessary for secondary metabolite gene cluster activation (Bok & Keller, 2004; Perrin et al., 2007). Also, volatile‐mediated suppression of *brlA*‐dependent sporulation may have caused the lack of many secondary metabolites that are specific to conidial tissue (Lim, Ames, Walsh, & Keller, 2014). It has been shown that the tissue of reproductive structures and resting stages of fungi contain specific and/or higher amounts of insecticidal compounds (Calvo & Cary, 2015; Döll et al., 2013). Morphological differentiation may thus be a general indicator of the defensive status of fungi. Our finding that microbial volatiles suppressed the formation of presumably insecticidal secondary metabolites, emericellamides and austinols upon insect damage (Caballero Ortiz et al., 2013; Döll et al., 2013) additionally suggests that key elements of the inducible defense of *A. nidulans* were affected. The correlation of the volatile‐mediated loss of insecticidal properties with the global reduction in mold secondary metabolites is consistent with our earlier findings: *Aspergillus* mutants hampered in the biosynthesis of secondary metabolites are less harmful to arthropods and are more susceptible to insect damage (Rohlfs, Albert, Keller, & Kempken, 2007; Caballero Ortiz et al., 2013; Trienens et al., 2010). Our results therefore underscore the central role of fungal secondary metabolism and the associated morphological changes in determining the capacity of a fungus to resist insect damage. But they also demonstrate the vulnerability of the fungal chemical defense system to the volatile means by which microorganisms engage in chemical interference competition (Cray et al., 2013; Schmidt et al., 2015).

From the insects' perspective, it is tempting to argue that microbial volatiles can create environments flies seek out actively to achieve protection from fatal molds. *Drosophila* flies, for example, may use the information contained in the volatile profiles of such environments as reliable cues for suitable breeding sites. Drosophilid flies are well known for their ability to discriminate between different yeasts and environmental contexts based on volatile information (Becher et al., 2012; Palanca, Gaskett, Günther, Newcomb, & Goddard, 2013; Günther, Goddard, Newcomb, & Buser, 2015). Attraction to yeast volatiles has been suggested to play a role in establishing mutualism‐like yeast–*Drosophila* associations (Buser, Newcomb, Gaskett, & Goddard, 2014), while nutritional benefits for the insects are thought to be the main driver of such associations (Anagnostou et al., 2010). Our finding that yeast volatiles interfere with the molecular mechanisms of secondary metabolite regulation in otherwise insecticidal molds adds another layer of complexity to yeast‐mediated “niche construction” and possibly the facilitation of (defensive) yeast–*Drosophila* mutualisms (Buser et al., 2014; Stamps et al., 2012). In this context, Schiabor, Quan, and Eisen (2014) recently showed that adult *D. melanogaster* flies are specifically attracted to yeast producing ethyl esters, like ethyl hexanoate and ethyl octanoate. These ethyl esters are also characteristic of the mold‐affecting volatile profile of *S. cerevisiae* described in the present study. Compared to the other volatiles, they were in fact produced “de novo” or in much larger quantities on the sugar‐rich medium (see Figure 3). Testing whether these compounds have a dual function in reducing mold insecticidal properties and providing cues to insect vectors and hosts (Hillman & Goodrich‐Blair, 2016), which possibly contributes to the evolution of habitat‐specific insect‐microbe associations (Adair & Douglas, 2017), remains an exciting task for future investigations.

## 5 CONCLUSION

Our study revealed that microbial volatiles have the potential to disable the activation of fungal chemical defense mechanisms, by which they indirectly benefited a saprophagous insect under controlled experimental conditions. Transcriptomic data suggest that cAMP/PkaA‐mediated cellular signaling involved in regulating morphological and chemical differentiation is a key target of the effect of microbial volatiles on *A. nidulans*. Probably, it is the volatile‐caused dysregulation of this signaling cascade that hampered the insect‐induced activation of insecticidal defense pathways and rendered the volatile‐exposed mold phenotype more vulnerable to insect damage. High amounts of carbohydrates in the surrounding matrix appear to be an important habitat characteristic that facilitated the shift from an allelopathic to a benign mold phenotype in our fruit‐related model system. Given the omnipresence of fungus‐affecting volatiles in decomposer habitats (Hol et al., 2015), we propose that volatile‐mediated changes in fungal phenotypes are of relevance also for other invertebrates, for example, for the soil‐dwelling fungivorous fauna. Future studies should address the diversity of effects of volatiles on fungal phenotypes and how volatile‐mediated changes in fungal combative abilities—as determined by their secondary metabolism—feed back on fauna–fungus community organization and dynamics.

## CONFLICT OF INTEREST

The authors have no conflict of interest to declare.

## DATA ACCESSIBILITY

GC‐MS data for *Saccharomyces* volatiles and HPLC‐MS data for *Aspergillus* extracts may be found as Supporting Information. Other data are available from the Dryad Digital Repository: https://doi.org/10.5061/dryad.pc1q925

## AUTHOR CONTRIBUTIONS

SOC, MT, and MR designed the study. SOC performed the gene expression analysis. MT ran the fitness assays. KP and PK performed the metabolic profiling of *Aspergillus nidulans*, and GH the volatile analyses of *Saccharomyces cerevisiae*. SOC, KP, PK, GH, and MR analyzed the data. MR wrote the first draft of the manuscript, and all authors contributed to revisions.
