# Supplementary material for: Phenotypic responses to microbial volatiles render a mold fungus more susceptible to insect damage
Source: Ecol Evol. 2018 Apr 2;8(8):4328–39. doi: 10.1002/ece3.3978 (PMC5916272; doi:10.1002/ece3.3978)
Supplement: Supplementary file 1 [file ECE3-8-4328-s001.docx]

**Phenotypic responses to microbial volatiles render a filamentous fungus more susceptible to insect damage**

Silvia Caballero Ortiz, Monika Trienens, Katharina Pfohl, Petr Karlovsky,

Gerrit Holighaus, Marko Rohlfs

**Supplementary material**

**Supplementary Figure S1** Tripartite yeast-mould-insect interaction


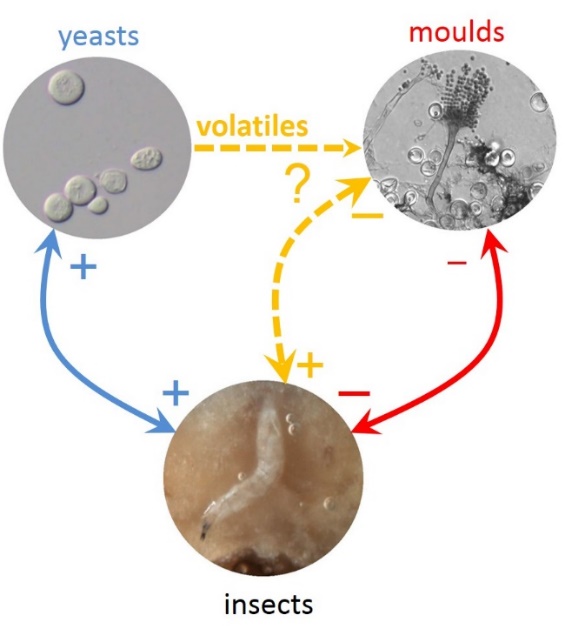


**Figure S1** Insect-yeast-mould interactions. Solid lines depict experimentally established relationships of Drosophila melanogaster larvae with yeasts and moulds. Yeasts support insect development, and larval activity promotes yeast growth. In bipartite interactions Drosophila larvae cause damage to mould colonies, but an inducible defence response renders the mould a fatal insect antagonist. The dashed lines depict a hypothetical scenario, in which yeast volatiles interfere with the mould’s insecticidal chemical defence mechanisms, and thereby change indirectly the outcome of this mould-insect interaction.

**Supplementary Figure S2** Reciprocal fungus-insect fitness consequences


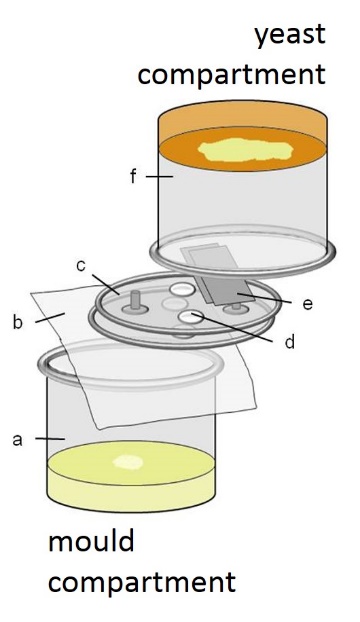


**Figure S2** Experimental set-up used for testing reciprocal fitness consequences in the *Aspergillus* *nidulans*-larval *Drosophila melanogaster* interaction under the influence of *Saccharomyces cerevisae* volatiles. Autoclavable round boxes (80131-7272, Bock Plastics Technologies, Lauterbach, Germany) with attached lids served as starting material. Each compartment (a, f) was 24 mm high and 25 mm in diameter. The two compartments were interconnected by fixing the lids with two stainless screws back-to-back (c). Two holes of 5 mm diameter (d) were punched out from the lids to allow diffusion of volatiles. The mould compartment was spanned with gauze (50 µm mesh size) (b) to prevent larvae from crawling into the yeast compartment. The holes in the connecting lids were additionally covered with a double-layer Miracloth® to prevent yeast cells from dripping and contaminating the mould compartment. Autoclaved compartments were filled with 2.5 ml sterilized medium each; banana medium in the mould and sucrose-supplemented malt-extract agar in the yeast compartment (see method description in the main text for more details).

**Yeast volatile-mediated effects on mould growth in different culture media**

with **Supplementary Figure S3**

To test whether the effect of yeast volatiles on the radial growth of *A. nidulans* is dependent on the culture medium, we conducted yeast-mould co-culture assays on three different media: standard *Drosophila* culture medium (62.5 g each of corn flour, yeast extract, and sucrose, 12.5 g agar, in one litre of tap water), banana fruit agar (finely crushed banana flesh mixed with the same volume of tap water, 15 g agar per litre banana-water mixture), malt-extract agar (30 g standard malt extract, 5 g soy peptone, 20 g agar in one litre of tap water). To distinguish the effects of yeast volatiles alone from any that might be caused by physical contact or diffusion of non-volatile compounds through the medium, we point-inoculated the mould (10 µl conidia suspension) and yeast (10 µl yeast cell suspension) on separate agar plates (3.5 cm diameter). One yeast- and one mould-inoculated plate were contained within closed 10 cm Petri dishes. Such experimental units with the respective yeast treatments plus yeast-free medium-only controls were replicated 20 times. After incubation at 25°C and 12 hours light cycle for 7 days, the plates were imaged. From the images, we recorded *A. nidulans* morphology and the area covered by mould using ImageJ 1.43u (Canon xc100s, http://imagej.nih.gov/ij). Yeast and mould culture medium significantly affected *A. nidulans* radial growth under the influence of yeast volatiles. On fly culture medium and banana agar, yeast volatiles strongly suppressed mould growth, whereas on malt extract agar no such effect was observed (Figure S3). Normal conidia development was observed in all yeast-free controls and in the yeast-malt extract treatment; no conidia were produced in the yeast-fly medium and the yeast-banana medium treatment (see Figure 2b and c). Note that adding sucrose to the malt extract agar restored the suppressing effect of yeast volatiles (see Figure S3). We thus conclude that comparatively high amounts of easily available carbohydrates, like in ripe fruits, contribute significantly to whether yeast volatiles have mould-affecting properties. Similar effects of sugars in producing anti-mould volatiles have been observed previously (Dzialo et al., 2017).

**
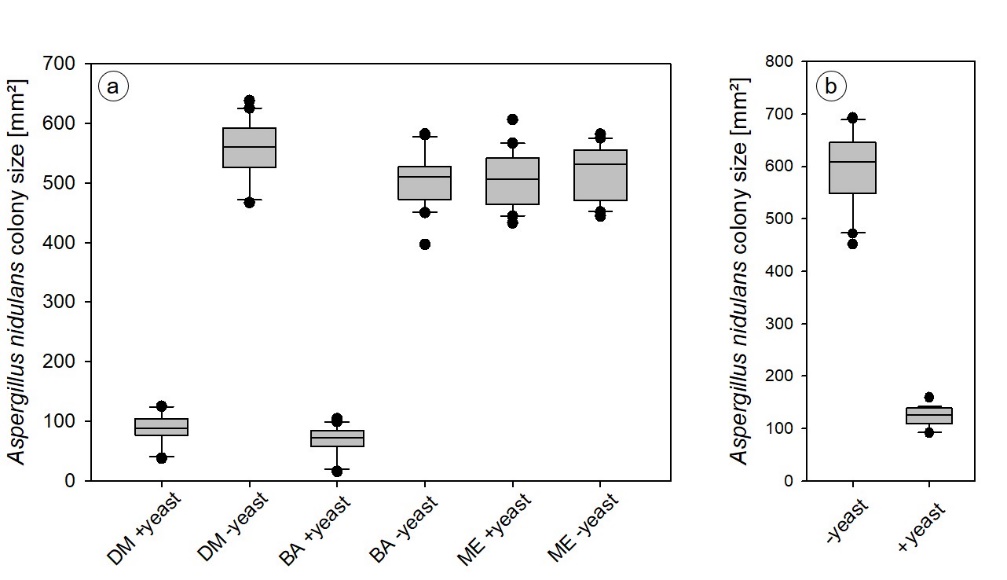
**

**Figure S3** Growth of Aspergillus nidulans on different culture media (a), and on malt extract agar supplemented with sucrose (b). Box-plots depict A. nidulans growth on different media (DM, Drosophila culture medium; BA, banana agar; ME, malt extract agar) when exposed to yeast volatiles, “+ yeast”. “– yeast” indicates mould growth without yeast volatiles. Solid horizontal lines indicate the median. (a) An ANOVA (Type III) revealed a significant effect of “medium” (F_2,114_= 4.71, p < 0.011), “presence of yeast” (F_1,114_= 1376.48, p < 0.001), and the interaction between these two main factors (F_2,114_= 441.69, p < 0.001). (b) The effect of yeast volatiles on ME agar plus extra sucrose revealed a similar effect on mould colony growth (F_1,37_ = 1779, p < 0.001). Colony size was square-root transformed prior to analysis.

**Specifications of the GC-MS analysis of yeast volatiles**

The GC–MS consisted of a 6890 N gas chromatograph connected to a 5973 N quadrupole mass spectrometer with electronic ionisation (EI, 70 eV; scan speed 2.25 full scans s-1), both Agilent (Santa Clara, USA). A nonpolar HP-5ms fused silica column (Agilent, 30 m, 0.25 mm ID, coating thickness 0.25 µm 5 % phenylmethylsiloxane) was used with a constant Helium flow of 1 ml min-1. Cryogenic oven-cooling with liquid nitrogen enabled good chromatographic separation resulting in high sensitivity and resolution for the yeast volatiles that have an overall low molecular weight (Watanabe-Suzuki et al., 2002): With an injection port held at 250 °C, the temperature program started at -30 °C (1.5 min), followed by an increase of 6.0 °C min-1 to 130 °C, thereafter with an increase of 30.00 °C min-1 to 200°C, hold for 3 min. Compounds were identified by comparison of full scan (m/z 20–345) mass spectra adjusted by a deconvolution software (AMDIS) and GC retention values with those of reference compounds and the mass spectral databases Wiley 9 combined with NIST 08 (McLafferty, 2009).

**Gene expression analysis**

Primers were designed using Primer Premier (Premier Biosoft) and synthesised and provided by Eurofins (www.eurofins.de): *laeA*_F: GCTCCTATTCAGCCTCCG, *laeA*_R: ATGACACTACCGCAACCC; *alfR*_F: GTCTCCGAATACTTCCACCT, *aflR*_R: ATGCCATCCATACCCTCA; *pkaA*_F: AACCACCGCTACTACTATGCC; *pkaA*_R: GGAAAGGATGCCTGACG; *brlA*_F: GTTGGCACGGAGCAGGAT, *brlA*_R: CGGGCATAGGCATTCG. For each sample, RNA was isolated from 100 mg lyophilised and thoroughly ground mould tissue. Reverse transcription of candidate RNA into cDNA and cDNA amplification was done in one step (One-Step qRT-PCR, Qiagen) on a Mx3000P qPCR System (Agilent) using SYBR Green (Lonza). PCR efficiencies (E), threshold fluorescence (R_CT_), and initial fluorescence (R_0_) were determined directly from the PCR kinetic curves using LinRegPCR (Ruijter et al., 2009). Fluorescence intensity as a measure of candidate mRNA quantity may vary for technical reasons. To control for that we normalised candidate RNA by applying an external luciferase control RNA spike (Promega L4561) (Rehrig et al., 2011; Caballero Ortiz *et al.*, 2013); primer sequences: *luc*_F: CCAGGGATTTCAGTCGATGT, *luc*_R: AATCTGACGCAGGCAGTTCT. For statistical analysis, we used normalised R_0_, equivalent to the initial amount of candidate mRNA in a sample (Schefe *et al.*, 2006), to compare the expression of *A. nidulans* genes in volatile-exposed and unexposed control colonies by means of a multivariate ANOVA on ranks (Caballero Ortiz et al., 2013).

**Supplementary Figure S4** Reciprocal fungus-insect fitness consequences

**

**Figure S4** Exemplary GC-MS total ion chromatograms of *Saccharomyces cerevisiae* volatiles (peaks pointing upwards) and the respective medium (mirrored downwards) either with supplemented sucrose (upper graph) or no sucrose (lower graph). Identical y-scaling, arbitrary unit - for details see Supplementary Table S1.

**Supplementary Figure S5** Absence of austinol in volatile-exposed *A. nidulans*

*
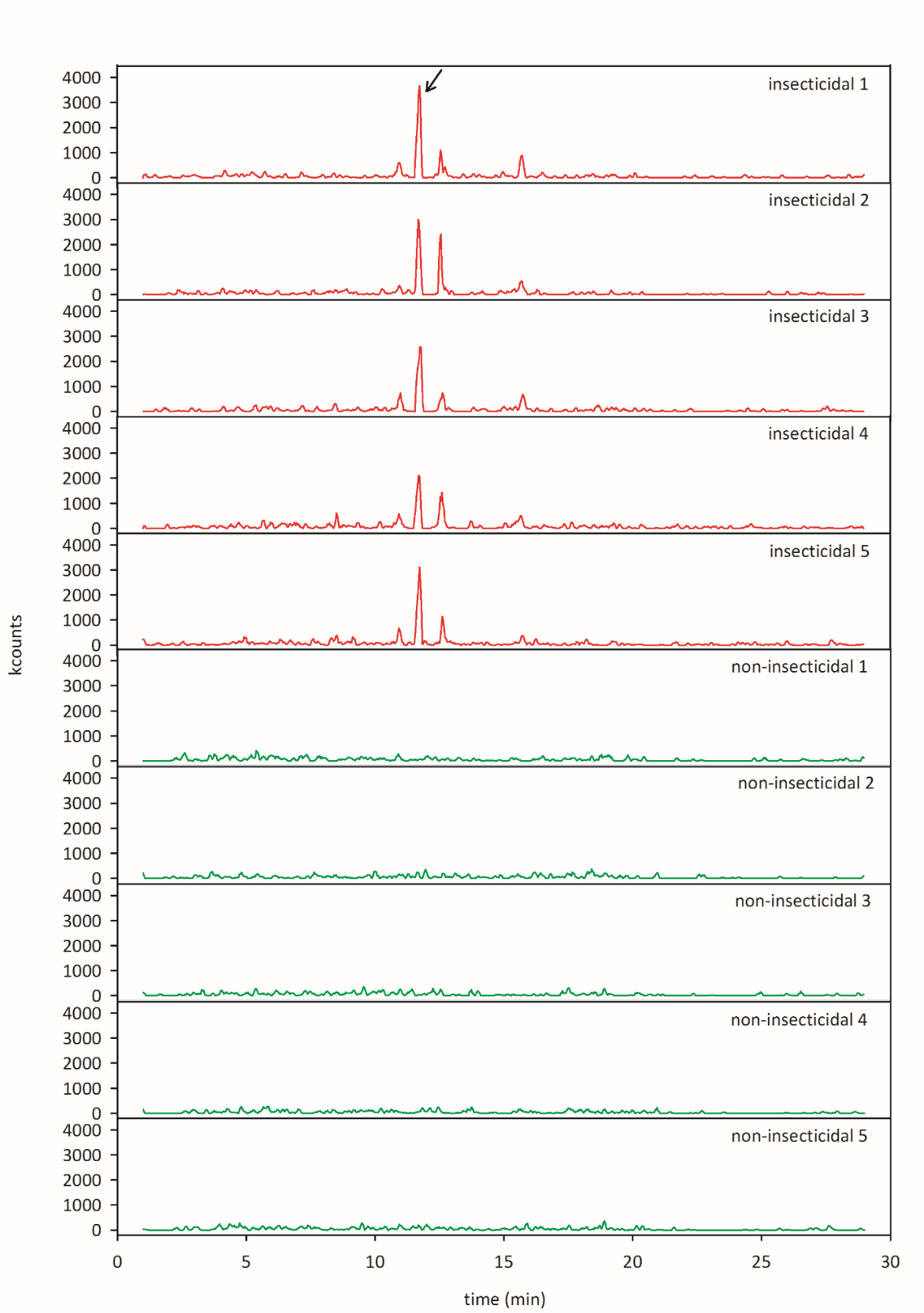
*

**Figure S5** LC-MS/MS chromatograms of austinol produced by unexposed/insecticidal (red) and volatile-exposed/non-insecticidal (green) *Aspergillus nidulans*. The arrow indicates the peak of austinol, whose identity was confirmed by two mass transitions, in which m/z 459>441 is shown here.

**References**

Caballero Ortiz S, Trienens M, Rohlfs M. (2013). Induced fungal resistance to insect grazing: reciprocal fitness consequences and fungal gene expression in the *Drosophila*-*Aspergillus* model system. *PLoS One*, 8, e74951.

Dzialo MC, Park R, Steensels J, Lievens B, Verstrepen KJ. (2017). Physiology, ecology and industrial applications of aroma formation in yeasts. *FEMS Microbiology Reviews*, 41, S95-S128.

McLafferty F. (2009). *Registry of mass spectral data combined with NIST/EPA/NIH database 2008*. Hoboken: Wiley-Blackwell.

Rehrig EM, Appel HM, Schlutz JC. (2011). Measuring ‘normalcy’ in plant gene expression after herbivore attack. *Molecular Ecology Resources*, 11, 294-304.

Ruijter JM, Ramakers C, Hoogaars WMH, Karlen Y, Bakker O, van den Hoff MJB, Moorman AFM. (2009). Amplification efficiency: linking baseline and bias in the analysis of quantitative PCR data. *Nucleic Acid Reseacrh*, 37, e45

Schefe JH, Lehmann KE, Buschmann IR, Unger T, Funke-Kaiser H. (2006). Quantitative real-time RT-PCR data analysis: current concepts and the novel “gene expression’s CT difference” formula. *Journal of Molecular Medicine*, 84, 901-910.

Watanabe-Suzuki K, Ishii A, Suzuki O. (2002). Cryogenic oven-trapping gas chromatography for analysis of volatile organic compounds in body fluids. *Analytical and Bioanalytical Chemistry*, 373, 75-80.
